# Supplementary material for: Metamachines of pluripotent colloids
Source: Nat Commun. 2021 Nov 4;12:6398. doi: 10.1038/s41467-021-26699-6 (PMC8569212; doi:10.1038/s41467-021-26699-6)
Supplement: Supplementary file 1 — Supplementary information. [file 41467_2021_26699_MOESM1_ESM.pdf]

Supplementary Information for : Metamachines of Pluripotent Colloids

Antoine Aubret,[1,2*] Quentin Martinet,[1,3] Jeremie Palacci[1,3*]

[1]Department of Physics, University of California San Diego
[2]Current Address: Univ. Bordeaux, CNRS, LOMA, UMR 5798, F-33405 Talence, France
[3]Current Address: IST Austria, Am Campus 1, 3400 Klosterneuburg, Austria

Contents

1 List of colloidal metamachines **3**

2 Synthesis of particles **4**

2.1 Hematite 4

2.2 Heterodimers 4

3 Sample preparation **5**

4 Dynamics of the swimmers under homogeneous illumination **6**

5 Hybrid architectures **7**

6 Optical setup **7**

7 Image acquisition and analysis **8**

8 Empirical determination of $\mathrm{M_S}$ **9**

9 Calculation of the optical field **10**

9.1 Analytical calculation 10

9.2 Numerical calculation 11

9.3 Comparison of optical forces 11

10 Optical forces on a hematite superball particle **12**

11 Motion of a self-propelled heterodimer entering an optical trap - simulations **13**

12 Computation of the phase diagram **16**

12.1 General procedure 16

12.2 Phase diagrams for different sizes of particles 16

13 Characterization of the laser profile **18**

13.1 Experimental determination 18

13.2 Simulations 19

14 Characterization of the trap stiffness **19**

14.1 Experimental determination 19

14.2 Simulations 19

15 Optical forces and torques on hemispherical janus particles **20**

16 Analysis of the angular statistics of swimmers in slender structures, effect of hydrodynamics **23**

17 Trapping requirements and alternate swimmers **25**

1 List of colloidal metamachines

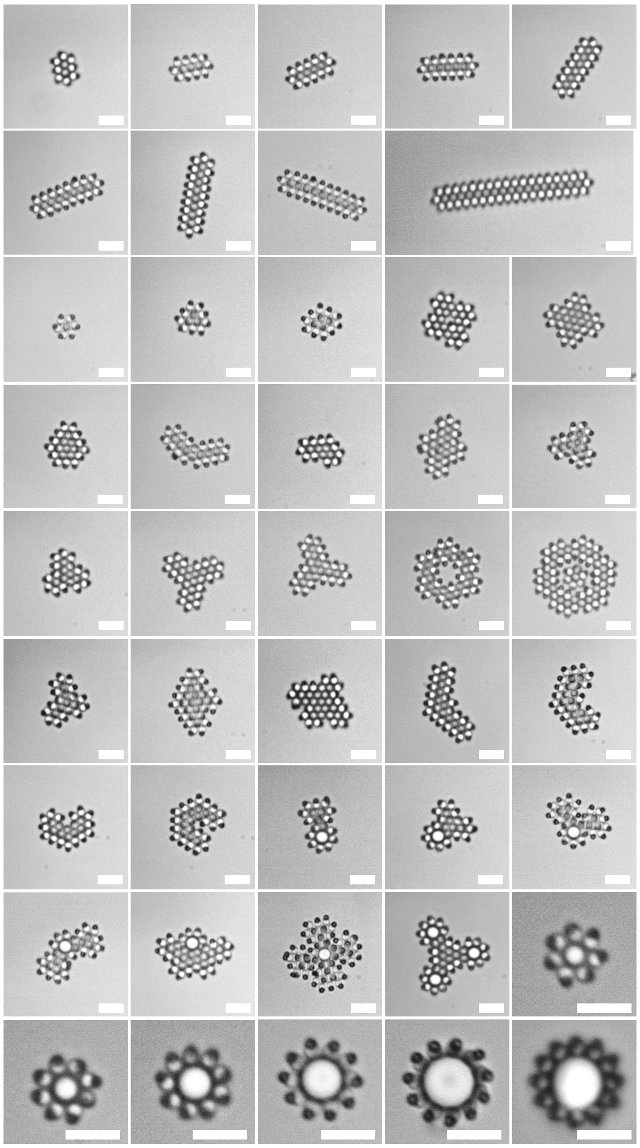

Supplementary Fig. 1: **List of Colloidal Metamachines :** Bright field images of the 44 metamachines assembled and studied for the present work. All scale bars are $5 \mu m$.

2 Synthesis of particles

2.1 Hematite

The synthesis procedure of hematite cubes follows the sol-gel method described by Sugimoto [1]. Briefly, we mix 100 mL of 2M $FeCl_3 \cdot 6H_2O$, 90 ml 6 NaOH and 10 ml water, in a 250 mL pyrex bottle and shake thoroughly. Immediately after, the bottle is placed in an oven at 100°C, and aged for 3 to 4 days, until the hematite particles reach the desired size (growth is monitored by optical microscopy). The resulting hematite cubes in the gel network are isolated by successive sedimentation and resuspension cycles in DI water [Supplementary Fig.2a].

2.2 Heterodimers

The synthesis of heterodimer particles is performed by heterogeneous nucleation of trialkoxysilanes (oil precursors) on solid hematite particles as seeds, and the control of their wetting properties with the pH of the solution. The synthesis procedure is adapted from ref. [2], with chemical modification to reinforce the stability of the heterodimer under light illumination. In particular, we make use of a hydrophobic copolymer Hexadecyltrimethoxysilane (HTS) to chemically protect the bond between the hematite and polymer core against highly reactive hydroxil radicals generated during H_2O_2 consumption. A beaker with 100 mL of DI water is prepared, and mixed with 120 μL of a 50% NH_3 solution, giving a pH $\sim$ 10.5. The solution is kept under mild magnetic stirring. We add $\sim$ 1 mL of an aqueous solution of hematite particles, to get a slightly red-colored solution. Following, we add 100 μL of HTS, immediatly followed by 500 μL of 3-(Trimethoxysilyl)propyl methacrylate (TPM). The solution is then covered with parafilm, and let under mild stirring for $\sim$ 2h00. During this time, the HTS and TPM hydrolyse, and condense on the hematite particles, with strong wetting leading to their engulfment in the oil phase. After $\sim$ 2h00, the solution is turbid, as a result of the scattering of newly formed colloidal particles. We then add 2 mL of Pluronic F-108 solution (5% wt), and wait 2 mn. The

dewetting and extrusion of TPM from the hematite is performed by changing the pH of the solution to pH ~ 2.1, by adding 1.5 mL of 1M chloridric acid HCl. The solution is let under stirring for 3 mn, and diluted 4 times. We then carry out the polymerization by adding 50 mg of radical initiator Azobisisobutyronitrile (AIBN) to the solution and leave it under stirring for ~ 5 mn. The beaker is covered with an aluminium foil and placed in a pre-heated oven at $\sim$ 80 degrees Celsius for 2 hours. We let the solution cool down to room temperature, remove the excess solution above the sedimented particles, and add 50 mL DI water with 1 mL of 250 mM NaOH solution, giving a pH $\sim$ 10, and let the solution overnight to facilitate the hydrolysis and condensation of any remaining HTS monomers. The solution is then centrifuged and rinsed multiple times to remove the excess TPM/HTS particles and obtain the desired colloidal solution of heterodimers [Supplementary Fig.2b].

3 Sample preparation

The samples are prepared at low particle density of $\sim 10^{-3}\ \mu\text{m}^{-2}$. The heterodimer particles are diluted in a 6% solution of hydrogen peroxide H_2O_2 (Fisher Scientific H325-500) in deionized water (Milli-Q, resistivity 18.2M). The cell for the solution is composed of a rectangular capillary glass (VitroCom 3520-050), previously subjected to plasma cleaning (Harrick Plasma PDC-001) and thoroughly rinsed with DI water. The solution is injected in the capillary, then sealed with capillary wax (Hampton Research HR4-328). As the particles are non-buoyant, they sediment near the bottom surface of the capillary, and observation with the microscope is made in this plane.

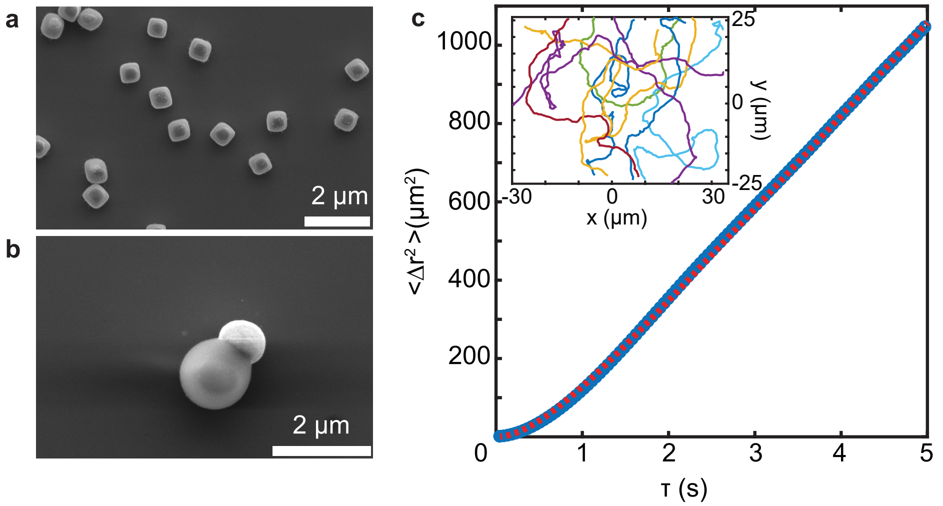

Supplementary Fig. 2: **Characterization of the particles :** Scanning Electron Microscopy (SEM) images for **a.** hematite cubes and **b.** the heterodimer particles used in this work. **c.**. Averaged Mean Square Displacement (MSD, blue circles) for the swimmers under blue light illumination only. The red dashed curve is a fit using eq.(1), fixing $D_T = 0.3$ μm^2/s, and giving $\tau_r = 1.1$ s and $v_S = 14.6$ μm/s as fitted parameters. *Inset :* typical trajectories of the swimmers, showing persistent random walk dynamics.

4 Dynamics of the swimmers under homogeneous illumination

Under constant blue illumination, the heterodimers exhibit persistent random walk, and both their velocity v_S and persistence time τ_r are extracted from analysis of their 2D mean square displacement $< \Delta r(\Delta t)^2 >$, which follows [3] :

$$< \Delta r(\Delta t)^2 >= 4D_T\Delta t + \frac{v_S^2\tau_r^2}{2}[\frac{2\Delta t}{\tau_r} + e^{-2\Delta t/\tau_r} - 1] \tag{1}$$

From fitting of the curve in [Supplementary Fig.2c], we find $\tau_r = 1.1$ s and $v_S = 14.6$ μm/s.

5 Hybrid architectures

Hybrid architectures composed of passive and active beads (see [Supplementary Fig.1]) are built using the same optical templating method as described in the main text. Passive beads are trapped at their center, and surrounded by an integer number of individual traps for microswimmers. For beads surrounded by 7, 8, 9, 10, or 11 swimmers, we used pluronic F108 coated TPM beads, synthesized as described in Ref. [4]. For beads surrounded by 12 or 13 swimmers, we used commercially available 7 and 8 μm polystyrene beads (Sigma Aldrich).

6 Optical setup

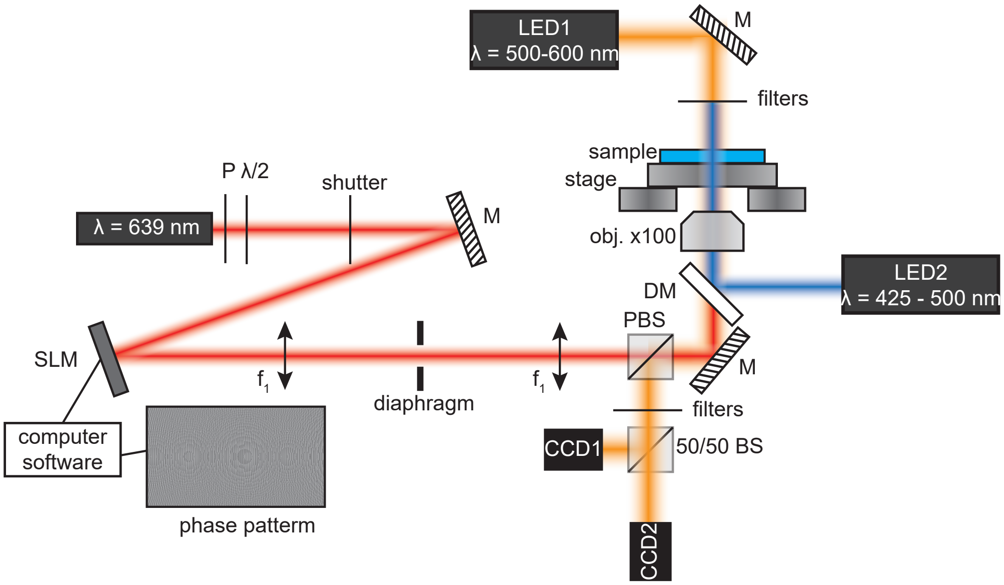

Supplementary Fig. 3: **Optical setup.** For better clarity, not all optical elements are represented. The letters stand for : P - Polarizer, $\lambda/2$ - half-waveplate, M - Mirror, SLM - Spatial Light Modulator, PBS - Polarizing Beam Splitter, BS - Beam Splitter, DM - Dichroic Mirror.

The experiments are carried out on a custom-made optical setup, allowing for simultaneous uniform excitation of the microswimmers and holographic optical tweezing, as presented on [Supplementary Fig.3]. The sample is observed with bright-field transmitted illumination

(LED1). A LED is set up in the blue range (LED2, $\lambda = 425 - 500$ nm, Lumencor SOLA 6-LCR-SC) and uniformly illuminates the sample on a large area (size $\sim 300 \times 300$ μm^2) to activate the swimmers by triggering the photocatalytic decomposition of H_2O_2 by the hematite (typical intensity of $\sim 1\,\mu$W/μm^2). A red continuous laser with near TEM00 mode ($\lambda = 639$ nm, Coherent, Genesis MX639-1000 STM, $M^2 < 1.1$) is added on the optical path. The linearly polarized beam is collimated, rotated with a $\lambda/2$ waveplate, and sent on the surface of a Spatial Light Modulator (SLM, Holoeye -LETO Phase Only Spatial Light Modulator). The optical path follows a typical 4-f setup using two $f_1 = 400$ mm lenses, and the zero-order of the diffracted beam is filtered out with a diaphragm at equal distance $d = f$ between the two lenses. Following, a hologram is formed at the back aperture of a high NA objective (Nikon apo-TIRF, $\times 100$, NA=1.45) allowing for the creation of complex spatiotemporal optical patterns in the object plane, at the bottom surface of the sample. The hologram is computed in real time using a computer software (Holoeye), with the phase patterns computed under Matlab, and allows for the selective trapping and manipulation of individual swimmers. An electronic shutter (Thorlabs SHB1T) on the red optical path enables switching ON and OFF the laser traps. The sample is mounted on a manual micrometric stage (Nikon Ti-SR). Observation is performed through the same objective as for excitation, and the bright-field signal is reflected on a polarizing beam splitter and observed on 2 monochrome Charged Coupled Devices (CCDs) with different resolutions (0.05 μm/px and 0.1 μm/px, respectively, Edmund Optics EO-1312M), with appropriate spectral filters.

7 Image acquisition and analysis

All experiments were recorded on the CCD camera with 0.05 μm/px resolution (except for Supplementary Movie 2 performed at lower magnification), at frame rate between 20-50 fps, and under bright field illumination. Tracking was performed separately for the hematite and

TPM particles with standard Matlab routine. For each of the 22 investigated structures, we record at least 10 s of experiments. The positions are then averaged a first time over 200 ms to compute $\omega(t)$, $\Omega_G(t)$, $\mathbf{v}_G(t)$; and further averaged to obtain 4 data points with equivalent statistical weight [Fig.3c - main text]. Error bars are obtained from standard deviations of experimental measurements.

8 Empirical determination of $\mathrm{M_S}$

We empirically extract and quantify the interaction of our swimmers with the surface from a statistical analysis of the angular position of the heterodimers [Supplementary Fig.4]. We record ≈ 12000 seconds of individual swimmers trajectories at a rate of 40 fps. For each trajectory, we independently track the positions of the hematite and TPM particles. The distribution of distances d between the two parts gives an approximate angular position as $\cos(\theta(t)) \sim d(t)/\max(d)$, assuming an orientation of the swimmer $\theta \in [0, -\pi/2]$. This gives the angular probability density function $P(\theta)$ at constant height, assuming that the swimmer does not significantly leave the wall over the course of the trajectory, i.e. $z/R_T \ll 1$, R_T being the radius of the TPM bead. From this, we extract the effective interaction potential with the surface as $V(\theta) = -k_b T \ln P(\theta)$, and $M_S(\theta) = -d_\theta V(\theta)$. The results show that the swimmer mostly slides with a tilt angle of $\theta_0 \sim -\pi/8$ above the surface, as a result of its effective interaction with the surface. The extracted torque holds for a swimmer velocity of $V_{S,0} = v_S/\cos\theta_0 \approx 16$ μm/s.

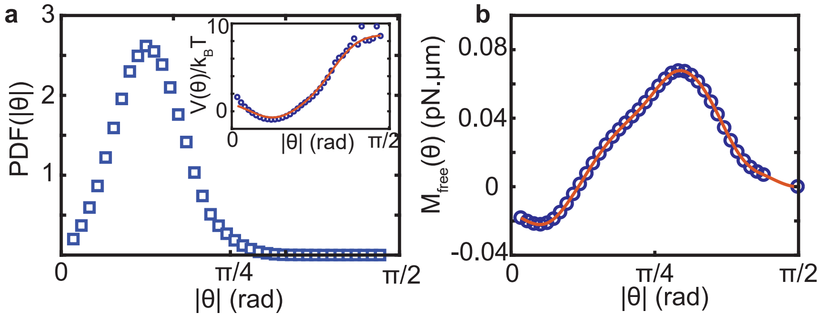

Supplementary Fig. 4: **Experimental determination of the torque acting on a swimmer near a surface.** **a**. Probability Density Function PDF($|\theta|$) of the measured angle of the microswimmers. It shows a clear peak at $|\theta| = \pi/8$, while the probability of having a free vertical swimmer ($|\theta| = \pi/2$) is close to zero. *Inset :* Extracted effective potential $V(\theta)/k_B T$. **b.** Plot of the torque $M_S(\theta)$, showing a maximum torque of ~ 0.08 pN$\cdot\mu$m at $|\theta| \sim \pi/3$.

9 Calculation of the optical field

9.1 Analytical calculation

The system is in the Mie regime, and uses a strongly focused laser beam (NA $\sim$ 1). We therefore use exact formulas for the optical forces acting on an a homogeneous sphere of any composition in the Mie-regime, formalized upon the Generalized Lorenz-Mie Theory (GLMT) for a highly focused incident gaussian beam in the angular spectrum representation. Knowing the full expressions of the scattered fields, the optical forces are obtained by integrating the Maxwell Stress Tensor over a surface surrounding the particle. The full expressions of F_x, F_y, and F_z and their complete derivation is described in Ref. [5]. We consider an incident beam at $\lambda = 639$ nm filling the entire back aperture of a water (refractive index $n_w = 1.33$) immersion objective, polarized along X and propagating along the Z-direction. We chose the parameters of the optics as to get a waist of $\omega_0 \approx 400$ nm that closely matches the experimentally determined value [Supplementary Fig.8]. The TPM part of the heterodimer is modeled as a non-absorbing dielectric bead of refractive index $n_T = 1.51$ and radius $R_T = 500$ nm, and the hematite part is modeled as a spherical body of radius $R_H = 250$ nm, homogeneously filled with $\alpha$$-$$Fe_2O_3$

($n_H = 3.10 + 0.065i$ at $\lambda = 639$ nm). The analytical expression, from Ref. [5], calculates the forces on individual TPM and hematite particles, that we use to approximate the total force on a heterodimer.

9.2 Numerical calculation

We use Comsol Multiphysics (v5.5) to compute the 3D optical field generated by a single, focused laser beam in presence of a *full* heterodimer particle in water. The solution for the background field is analytically approximated using the angular spectrum representation of the focal field for a non-paraxial beam, and using discrete summation to approximate the continuous integration over the numerical aperture of the focusing lens. We model infinite boundaries in our system using the Perfectly Matched Layers module of COMSOL, which prevents any reflection of the scattered field on our artificial boundaries. The optical forces and torques F_x, F_y, F_z, $M_{scat,xyz}$ are then obtained by integrating the Maxwell stress tensor on a surface surrounding the heterodimer [6]. We check the incident power by integrating the flux of the Poynting vector associated to the background field across the transverse XY plane. For single particles, the forces computed with COMSOL agree perfectly with the analytical expressions, for identical beam parameters.

9.3 Comparison of optical forces

In [Supplementary Fig.5a,b], we show the 2D mapping of the optical forces for single hematite and TPM beads in water, using analytical calculations. While the gradient force pulling the particle near the center of the focus dominates for the TPM part, the scattering forces are dominant on the hematite particle, pushing it away from the focal point of the laser. We now aim to compare the total optical force exerted on a [hematite-TPM] heterodimer, where the forces on the individual hematite and TPM particles are estimated analytically with the total force ex-

erted on the heterodimer using COMSOL calculations [Supplementary Fig.5c,d]. For a particle placed at the center of the incident laser beam, we observe negligible deviation between the torque computed by COMSOL and the torque estimated by summing the forces on each individual components and estimated analytically [Supplementary Fig.5c]. The forces computed along z departs more significantly in the two models, in particular at higher heights for a vertical heterodimer facing the substrate [Supplementary Fig.5d]. The lower magnitude of the force obtained at larger distance from the surface using COMSOL compared to the analytical calculations however suggests an increased trapping stability at higher power and does not weaken our conclusions (see main text, [Fig.2e] and [Supplementary Fig.7]). It follows that the total force acting on a heterodimer is well approximated by summing the isolated forces of its two components, and we therefore use this method for mapping of the optical forces in the $\{X, Z, \theta\}$ space.

10 Optical forces on a hematite superball particle

In this section, we investigate in more details the effect of the shape of the scattering component. We specifically compare the scattering forces that apply on the hematite component, as a near cubic shape of the hematite (a superball, as discussed in Ref. [7]) or a spherical hematite particle of comparable size. We consider a hematite superball of width 500 nm (see [Supplementary Fig.2a] and [Supplementary Fig.6a]) at distance x from the center of a focused laser beam in water, as described above. We perform Comsol simulations of the incident and scattered field, and compute the optical forces acting on the superball oriented with different angles relative to the optical axis [Supplementary Fig.6b]. The results are plotted for both F_x (dashed lines) and F_z (dotted lines) against the ones for a spherical particle of radius 500 nm (plain lines). We find that both F_x and F_z weakly depends on the inclination θ of the superball with respect to the incident light, making the scattering of the superball in the considered optical regime effectively

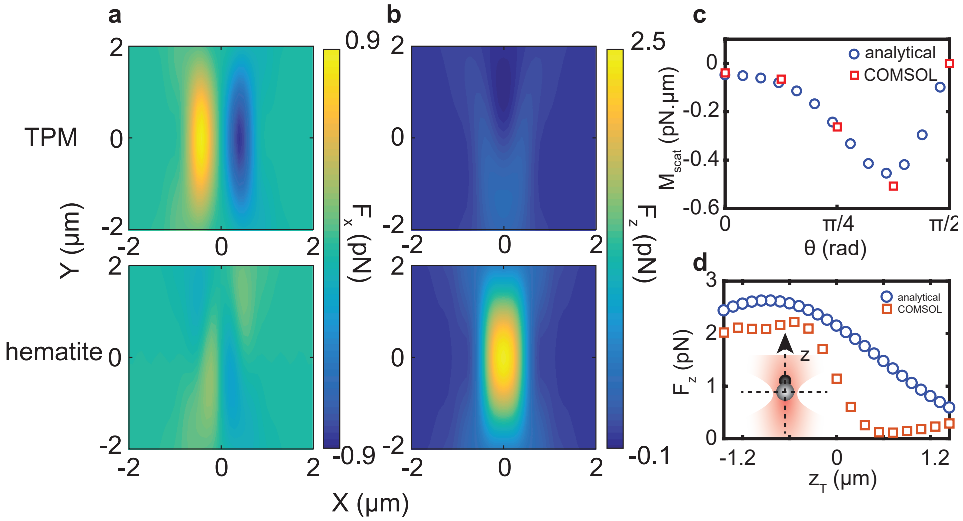

Supplementary Fig. 5: **Optical forces on the heterodimer.** **a**. Optical Forces along the X direction for single hematite ($R_H = 250$ nm) and TPM ($R_T = 500$ nm) particles. **b**. Optical Forces along the Z direction. For better comparison, colorbars are identical for both particles. **c**. Computation of the optical torque for a heterodimer with the TPM bead situated at the focus, as a function of the orientation θ. The sum of individual forces on each component in the absence of one another (analytical) is compared to COMSOL calculations considering a full heterodimer. **d**. Similar comparison as in (**c**), but for the force along Z acting on a vertical heterodimer, as a function of the position of the TPM bead. The scheme indicates the considered geometry. All forces and torques are computed for a 1 mW incident power.

similar to the scattering by a sphere. This result is confirmed by the fact that the forces for the superball quantitatively agree with the optical forces computed for a spherical particle (solid line). As a result, we did not consider the shape of the heterodimer and used spherical particles to capture the main features of the interaction of the heterodimer with the optical potential.

11 Motion of a self-propelled heterodimer entering an optical trap - simulations

We consider a simplistic model where the heterodimers are self-propelled particles in the absence of noise. Heterodimers are considered rigid bodies constituted of 2 distinct parts in the XZ plan, with orientation $\mathbf{u}_\theta$. The position $\mathbf{r} = \{x, y = 0, z\}$ is defined as $\mathbf{r} = [\gamma_H \mathbf{r_H} +$

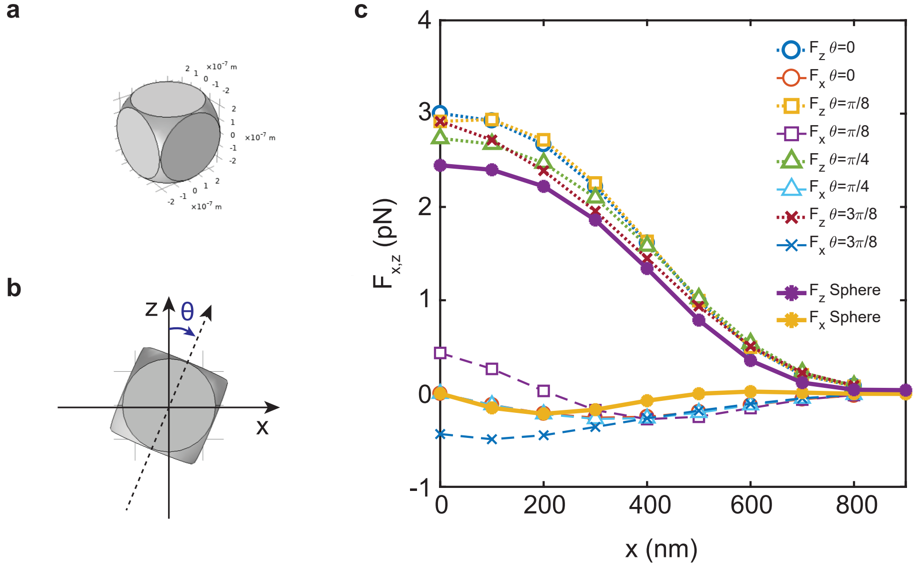

Supplementary Fig. 6: **Optical forces on a hematite superball.** **a.** Perspective view of the considered geometry for a superball of width 500 nm, as modeled with Comsol. **b.** Scheme of the orientation axis and representation of the angle of rotation θ of the cube around the y-axis. **c.** Numerical results for the forces along x (dotted lines) and z (dashed lines) for different orientations θ. The results are plotted for a hematite particle at $z = 0$ and varying x positions with respect to the center of the beam. Solid lines show the corresponding optical forces for a spherical hematite particle with diameter 500 nm.

$\gamma_T \mathbf{r_T}]/(\gamma_H + \gamma_T)$ to account for the different drag coefficients $\gamma_H = 6\pi\eta R_H$ and $\gamma_T = 6\pi\eta R_T$ acting on the hematite and TPM beads situated at $\mathbf{r_H}$ ad $\mathbf{r_T}$, respectively. In our model, we discard hydrodynamic interactions between the hematite and the TPM. The equations of motion are determined from balancing external forces and self-propulsion by the viscous drag, i.e. : $\sum \mathbf{F}_{ext} = \mathbf{F}_{drag}$ and $\sum \mathbf{M}_{ext} = \mathbf{M}_{drag}$.

The total drag torque is obtained as $M_{drag} = \Gamma\omega$, with $\Gamma = \gamma_H {R_T}^2 + \gamma_T {R_H}^2 + (4/3)(\gamma_H R_H^2 +$

$\gamma_c R_T^2$). For a swimmer with propulsion velocity V_S, we write :

$$\frac{dx}{dt} = V_S \cos(\theta) + \frac{\sum F_{ext,x}}{\gamma} \tag{2}$$

$$\frac{dz}{dt} = V_S \sin(\theta) + \frac{\sum F_{ext,y}}{\gamma} \tag{3}$$

$$\frac{d\theta}{dt} = \frac{1}{\Gamma} \sum M_{ext} \tag{4}$$

where $\gamma = \gamma_H + \gamma_T$.

We simulate the motion of particles in the presence of optical forces, acting independently on the particles, using finite-difference equations as derived from eq.(2-4). We consider the linear optical regime, such that the optical forces $F_{opt} \propto P_{inc}$, with P_{inc} the incident power. We model the substrate as an impenetrable wall situated at $z = -R_T$, such that any step for which the particle penetrates the wall yields to a displacement of the swimmer to be in close contact with the wall, i.e. we force $z_T \geq 0$, and $z_H \geq R_H - R_T$. We obtain $M_S(\theta)$ by interpolation from the experimental values, and apply the same procedure for the optical potential $M_{scat}(\mathbf{r}, \theta)$: we extract the forces applied on the hematite and TPM parts individually, allowing us to approximate both torques and forces $F_{scat,x,z}(\mathbf{r}, \theta)$ acting on the heterodimer.

The torque $M_S(V_S, \theta)$ depends on the instantaneous propulsive force (i.e velocity V_S) of the swimmer. In the Stokes regime ($Re << 1$), we can reasonably assume both phoretic and hydrodynamic effects to be linearly dependent on the velocity of the swimmer. Hence, we assume linear dependency of $M_S(V_S, \theta)$, such that $M_S(V_S, \theta) = (V_S/V_{S,0})M_S(\theta)$, where $V_{S,0} = 16$ μm/s is the velocity of the swimmer in free space. Finally, the gravitational torque is negligible compared to the typical values for M_{scat} and M_S, and the effect of the gravitational torque is therefore not included when computing the trajectories.

12 Computation of the phase diagram

12.1 General procedure

We vary both P_{inc} and V_S, and compute the trajectories followed by the heterodimers upon crossing the laser beam at zero noise. For all trajectories, the swimmer starts with the TPM part at $\{x_{T,0} = -1000nm, y_{T,0} = 0, z_{T,0} = 0, \theta_0 = -\pi/8\}$. We assume that stable trapping is achieved as long as the swimmer stays in the focus of the beam, corresponding to $-w_0 < x_T < w0$, and $0 < z_T < z_R$, where w_0 is the waist of the laser, and z_R the Rayleigh range, defined as $z_R = 0.5kw_0^2$, where k is the wavector in water. Crossing is achieved when the swimmer reaches $x_T \geq 2000$ nm, and is ejected when $z_T > z_R$. The equations of the dynamics are given by eq.(2-4). All simulated trajectories are performed for 10 s, with a time step of 5 ms. The various scenarii are summarized in [Supplementary Fig.7a].

12.2 Phase diagrams for different sizes of particles

The results for heterodimers with similar size compared to the experiments are presented in the main text [Fig.2e]. We extend our simulations to the case of heterodimers with various hematite and TPM sizes. In the absence of a measured experimental torque M_S (see section 8 of SI), we consider only gravitationnal forces to contribute a torque to the rotation of the heterodimer, on top of optical forces. The results are presented in [Supplementary Fig.7b-d] for 4 different sets of sizes, with beam waists of $\omega_0 = 420$ nm when $R_T = 500$ nm, and $\omega_0 = 240$ nm when $R_T = 250$ nm, in order to match the size of the beam focus with the TPM diameter. In all considered cases, we observe the three phases (crossing, trapping and ejection), with a larger trapping region in the $\{P, V_{SW}\}$ attained for smaller particles and beam waists. This shows the potential of the trapping method to be applied for various kinds of heterodimers and beam parameters.

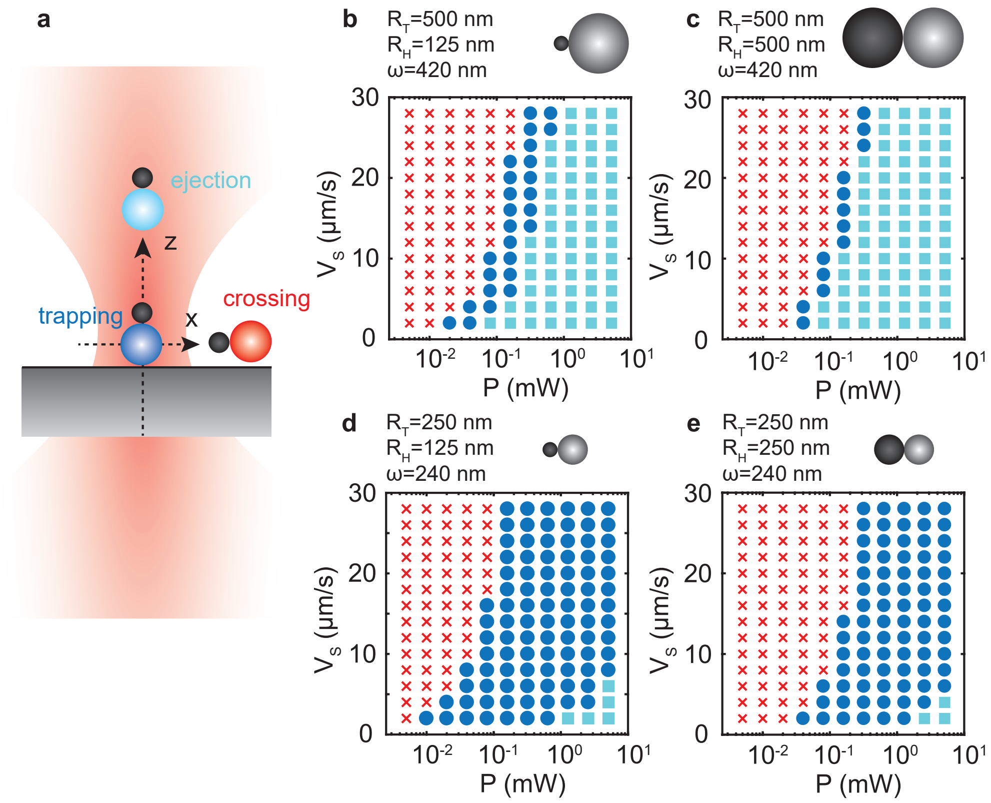

Supplementary Fig. 7: **Trapping phase diagram computation.** **a.** Scheme of the different scenarii for the trajectories of self-propelled heterodimers, in the absence of thermal noise. The heterodimers are ejected upward by radiation pressure (cyan squares), cross the laser spot (red crosses), or stay at the center of the beam (blue circles). **b-e.** Phase diagrams computed for various sizes of heterodimers : **b.** for $R_T = 500$ nm, $R_H = 125$ nm, and $\omega_0 = 420$ nm; **c.** for $R_T = 500$ nm, $R_H = 500$ nm, and $\omega_0 = 420$ nm; **d.** for $R_T = 250$ nm, $R_H = 125$ nm, and $\omega_0 = 240$ nm; **e.** for $R_T = 250$ nm, $R_H = 250$ nm, and $\omega_0 = 240$ nm.

13 Characterization of the laser profile

13.1 Experimental determination

We measure the width of the laser spot from the reflection of the laser beam on the glass/water interface, imaged on the CCD camera, at resolution of 0.03 μm/px [Supplementary Fig.8a]. Substracting background noise, the intensity profile is well fitted by a gaussian curve, from which we extract $\sigma = 210 \pm 10$ nm, which corresponds to a waist $\omega_0 \sim 2\sigma = 420$ nm.

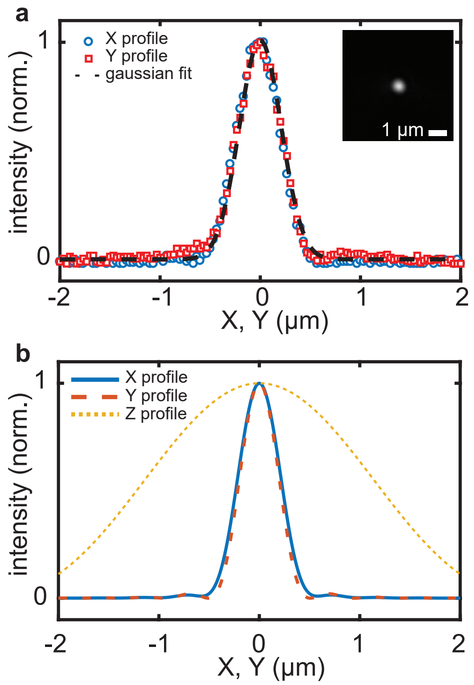

Supplementary Fig. 8: **Laser beam profile.** **a**. Experimental measurement of the intensity profile at the focus, obtained from the reflection of the laser beam on the glass/water interface, in both the X and Y directions. The dashed line is a fit of the data points with a gaussian function, giving a standard deviation $\sigma = 210$ nm. *Inset :* Image of the reflected laser spot. Scale bar is 1 μm. **b**. Intensity profile in the X, Y, and Z direction for a beam propagating along the Z direction and polarized along X, calculated using the angular spectrum representation of a focused laser beam, at the focus. Fitting with a gaussian the profiles in the transverse plane gives a similar waist as $\sigma = 200$ nm.

13.2 Simulations

We use the angular spectrum representation of a strongly focused laser beam to compute the optical forces acting on the particles (hematite and TPM). In all analytical and numerical computions, we consider a TEM00 incident gaussian beam mode polarized along x and propagating along z, with an apodization function equals to 1, which corresponds to a flat beam profile at the back aperture of the focusing lens . The profile of the simulated laser beam in water, as presented in [Supplementary Fig.8b], agrees well with the experimentally determined one.

14 Characterization of the trap stiffness

14.1 Experimental determination

We measure the optical force acting on a TPM bead only ($R_{TPM} \approx 0.6$ μm), at equilibrium, by trapping it and measuring the position fluctuations for different powers of the incoming beam. At equilibrium, the restoring force of the optical trap competes with thermal fluctuations, and the stiffness of the trap is given by : $k_{x,y} = k_B T / < \Delta r^2_{x,y} >$. The probability density function PDF(x,y) is well approximated by a gaussian curve with standard deviation $\sigma^2_{x,y} =< \Delta r^2_{x,y} >$ [Supplementary Fig.9a]. Fitting the normalized histogram of positions, we extract the stiffness for various intensities, and typically get $k = 0.8 \pm 0.1$ pN/μm at 0.5 mW incident power, with a linear power dependency of the trap stiffness, and a slope of ≈ 1.7 pN/(μm.mW).

14.2 Simulations

Following the calculation of the force, we simulate the 1D trajectories of a single spherical TPM particle ($R_T = 500$ nm, $n_T = 1.51$) in water ($n_w = 1.33$), in presence of the focused laser beam. The trapping stiffness is measured as described in the previous section 14.1. The results, presented in [Supplementary Fig.9b], show good agreement with the experimental values, with a slope of ≈ 2.2 pN/(μm.mW).

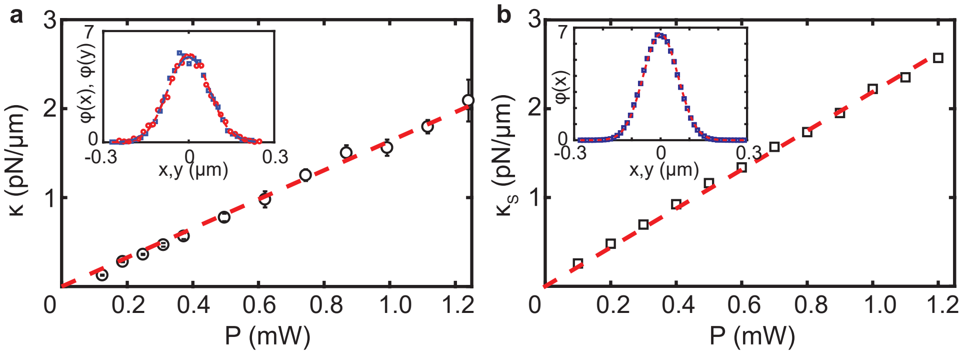

Supplementary Fig. 9: **Experimental characterization of the trap stiffness for a single TPM bead. a**. Experimental measurement of the stiffness of the trap, determined for various powers of the incoming beam, and fitted by a linear function (red dashed curve), giving a slope of 1.7 pN/(μm.mW). Error bars are standard deviations from multiple experiments. *Inset :* Example of the PDF(x,y) of the position of the bead in the trap at 0.5 mW incident power for both x (blue squares) and y (red circles) coordinates. The PDFs are perfectly fitted by gaussian curves. **b**. Stiffness of the trap extracted from simulated trajectories of trapped $R_T = 500$ nm TPM beads, showing similar trend as experimental data. *Inset :* PDF(x) of the bead at 0.5 mW incident power.

15 Optical forces and torques on hemispherical janus particles

We demonstrate the generic nature of activity-enabled optical trapping to common janus microswimmers, half coated by a nanometric metal film. Such janus microswimmers can be produced to propel without photocatalytic activity, such as Pt-coated janus particles in H_2O_2[8, 3], or exploiting light induced thermal effects as demonstrated for Au-coated janus particles [9, 10, 11].

As such, we explore the effect of optical forces on both Au and Pt coated janus particles. In each case, we use a simple model of a core shell spherical particle of total radius 500 nm and variable shell thickness between (10-80 nm), immersed in water. Half of the shell is made of metal, while the rest is made of silica [Supplementary Fig.10a]. We consider an incident beam as described previously, propagating along the z-direction. In all cases, the particle is at

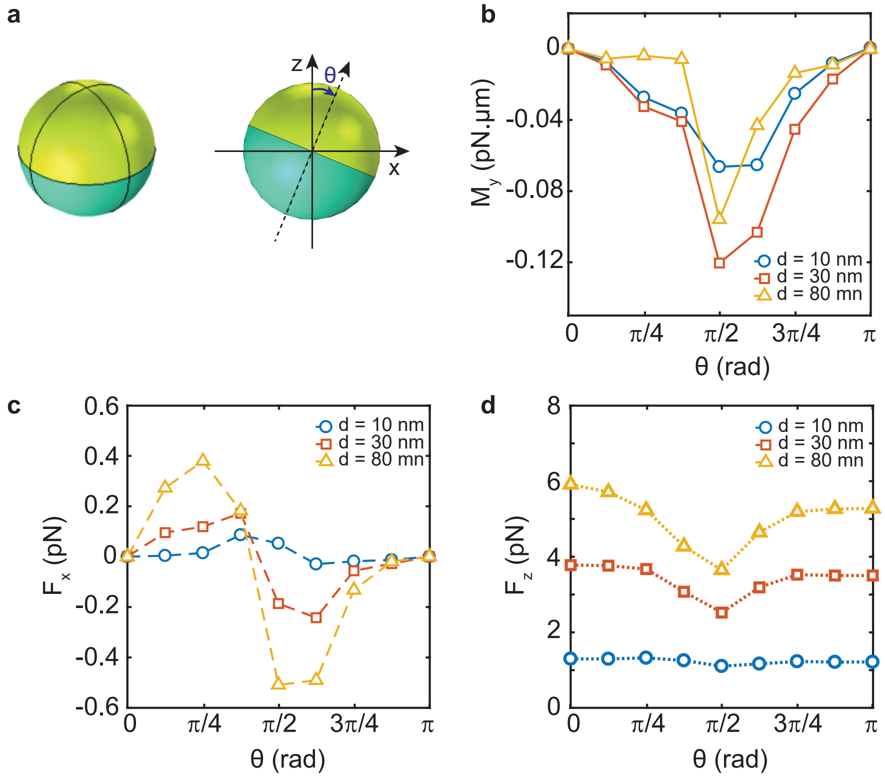

Supplementary Fig. 10: **Optical forces and torques on a hemispherical Au-coated janus particle.** **a.** Scheme showing the considered geometry : a core shell particle has half of its shell constituted of a gold part (yellow). We compute the optical forces and torques acting on it for various angles θ with respect to the optical axis, the metal part pointing up at $\theta = 0$, with an incident beam of $P = 1$ mW, $\lambda = 532$ nm, and $\omega_0 \approx 400$ nm. **b.** Optical torque M_y on the full janus particle, for 3 different thicknesses, **c.** F_x and **d.** F_z.

the center of the laser beam. We compute the scattered field using Comsol and integrate the Maxwell stress tensor over an arbitrary surface surrounding the particle to obtain both forces and torques acting on the particle, varying the orientation θ of the janus with respect to the optical axis z [Supplementary Fig.10a].

We first present the results for a Au janus particle (see [Supplementary Fig.10b-d]) having three different Au thicknesses of 10, 30, and 80 nm. The incident power is $P = 1$ mW, the wavelength is $\lambda = 532$ nm, and the waist is $\omega_0 \approx 400$ nm. We observe a negative optical torque

that aligns the particle with the optical axis, presenting a maximum value at $\theta = \pi/2$. The graph for F_x shows a reversal of the sign of the force with orientation, while F_z stays positive. Both forces grow monolitically in amplitude with shell thickness, indicative of a concomitant increased scattering force.

We conduct identical simulations for a 10 nm Pt-covered spherical particle, as presented in [Supplementary Fig.11], under 1 mW incident power at $\lambda = 632$ nm, and for a waist of $\omega_0 = 420nm$. The results show similar trends as for an Au particle, with higher upward scattering force generating higher optical torque for comparatively smaller thickness. Notably, while the torque shows a more complex signature, its negative value ensures a realignement with the optical axis.

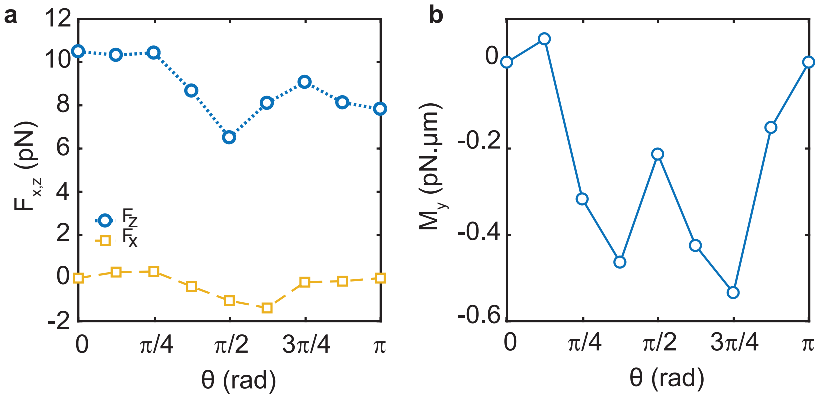

Supplementary Fig. 11: **Optical forces and torques on a hemispherical Pt-coated janus particle.** The considered geometry is identical to the one presented in [Supplementary Fig.10a], replacing Au by a 10 nm Pt cap, and with an incident beam of $P = 1$ mW, $\lambda = 632$ nm, and $\omega_0 \approx 420$ nm. **a.** Forces F_x and F_y for various orientations of the janus, at the center of the beam. **b.** Optical Torque M_y acting on the janus. All data show similar trend as for the Au coated janus particle.

The effect of asymmetric scattering on the motion of spherical janus particles has been studied before, mostly at the individual particle level [12, 13, 13, 14, 15]. Our numerical results are in qualitative agreement with those previous studies, showing that an orientation dependent optical torque of similar magnitude appears and can be modulated by the material properties, or the

parameters of the incident beam such as the wavelength and power. For a passive (i.e. without self-propulsion) particle, the equilibrium position and orientation in the trap may differ from the center of the beam [13], as evidenced by the non-zero transverse forces that we obtained with our numerical simulations. The addition of a propulsive force opposing the scattering may offer new opportunities for the 3D manipulation and precise positioning of active particles. This aspect has been approached at the single particle level [16, 17, 18], but remains to be explored for larger assemblies of janus particles. Overall, our results suggest that activity-enabled optical trapping can used with hemispherical Janus particles, enabling the use of algorithms of formation of machines. It highlights the broad applicability of the approach, not requiring light-activated particles nor a specific mechanism for propulsion.

16 Analysis of the angular statistics of swimmers in slender structures, effect of hydrodynamics

The persistence of the motion of an architecture depends on the fluctuations of orientations of the constitutive swimmers. In order to gain insight on the influence of hydrodynamics on the angular distribution of the heterodimers with $n = 3, 4$ neigbors, we investigated the dynamics of slender structures of various lengths, that we use as model systems [Supplementary Fig.12]. These rod-like structures exhibit primarily rotational motion around their barycenter, with a rotational speed dependent on the length of the structure. In addition, in those structures the heterodimers at the tip of the rods have a constant nearest-neighbors environment, independant of the length of the rod-size, and experience a hydrodynamic drag in similar direction. It allows us to investigate the effect of rotation speed of the machine on the orientational dynamics of the heterodimers with $n = 3, 4$ at the tip, and quantify the effect of hydrodynamics. We study the dynamics of elongated rods ranging from $N = 7$ to $N = 31$ swimmers [Supplementary Fig.12a]. The results, presented in [Supplementary Fig.12b], show a broadening of the angular

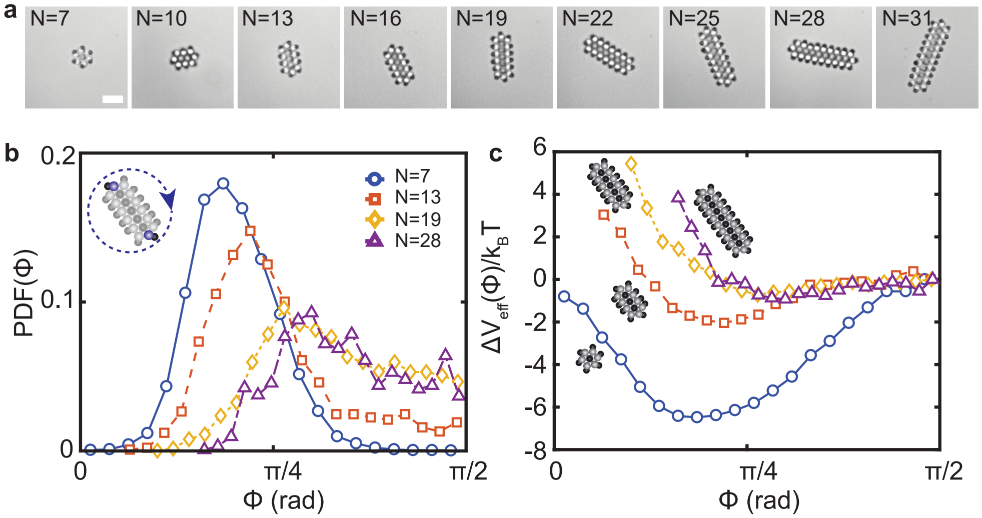

Supplementary Fig. 12: **Analysis of the orientation of swimmers in slender structures.** **a**. List of slender structures that were investigated, with corresponding number of swimmers N. **b**. Histogram of the orientation Φ of the heterodimers situated at the tips of the structures. Upon increasing the size of the structures, the PDF(Φ) broadens, and the orientation of the heterodimers can flip direction. The scheme for a $N = 19$ structure shows the investigated 'tip swimmers' in blue, together with the sense of rotation (blue arrow). **c**. Corresponding effective potential, shifted so that $V(\pi/2) = 0$. The difference in potential depth is clearly visible, and is less than $k_B T$ for large rods with $N \gtrsim 16$.

distribution for increasing rod size and subsequent decreasing rotational velocity. It highlights the role of hydrodynamics on the orientational dynamics of the swimmers, coupled to collective motion of the metamachine. We estimate an effective hydrodynamic potential from the distribution of angles, assuming that Boltzmann statistics remain valid, a questionable choice out of equilibrium but allowing us to get a physical intuition of the strength of the effect. We obtain an effective potential $V(\Phi)/k_B T \approx -\ln PDF(\Phi)$ [Supplementary Fig.12c], and presenting a well defined minimum $\sim 6k_B T$ for 7-swimmers rods, vanishing to $\sim 1k_B T$ for rods larger than ~ 16 swimmers. It highlights the "stabilization" effect of hydrodynamics in the orientational dynamics of the heterodimers pushing on the edge. As a result, large and slow structures are more likely to be dynamically metastable, and switch between different dynamics.

17 Trapping requirements and alternate swimmers

We emphasize that the fact that the self-propelled particles used in the present work are light-activated is irrelevant for the trapping mechanism. In fact, different wavelengths are used for activation (blue light) or trapping (red light) of the particles. With a bandgap of $2.1eV$, the photocatalytic activity of hematite above 590nm is negligible, making possible to consider the effects of each wavelength independently. For the purpose of the present work, we use uniform blue light, resulting in spatially uniform activation and propulsion. It results that under uniform blue light, our light-activated particles exhibit a generic persistent random walk with constant velocity V. They behave as generic, non light-activated, self-propelled particles. Our model and proposed algorithm of formation of machines only requires self-propulsion and particles that experience an aligning torque due to optical forces, as is often the case for anisotropic particles. Such requirements are fairly common features for active colloids, that are often anisotropic to achieve propulsion. This is for example the case for the widely used Janus microswimmers, coated on one side with a metal cap. It follows that our conclusions are applicable to a broad class of self-propelled particles and we summarize, here, minimal requirements for alternate swimmers.

The main requirement for activity-assisted optical trapping is an adequate balance between propulsion and optical forces. The strength and direction of the optical force will indeed depend on the specifics of the dielectric and geometric properties of the active particles but adequate optimization of laser parameters is possible to enable optical trapping. We have confirmed this qualitative statement by performing additional computations (i) of dimers of with different ratio of size between hematite and main body, (ii) spherical Janus microswimmer [Supplementary Fig.7, 10 and 11]. In addition, transverse gradient force are helpful to increase the trapping

stability of the swimmers, and requires the index of refraction of the particle n_p to be larger than the one of the medium n_m.

For experimental implementation, the two main ingredients are the following : 1) an asymmetric scattering leading to reorientation of the particle in the light field, and 2) the presence of optical gradient forces in the transverse plane to stabilize the trapping of the particle near the center of the beam. As scattering forces on metal caps, as for Janus particles, are repulsive, it requires the self-propulsion to direct against the incident light. Effectively, it requires a direction of propulsion of the particle away from the scattering portion of the particle. The determination of the direction of propulsion of self-propelled particles can be finicky to define *a priori*, as it is phoretic in nature and can depend on details of the particle solid surface [19]. On the other hand, this interfacial nature also provides means of control to set the direction of propulsion. The addition of surfactants [20] or pH change [21] of the suspending medium were shown to alter the direction of propulsion of self-propelled particles, thus emphasizing the potential of surface properties alteration to control propulsion direction. Using diffusiophoretic self-propelled particles (silica particles capped with carbon suspended in a critical binary mixture), control of the velocity and orientation of the particle was achieved using uniform green light illumination [22]. Finally, we also want to mention that in the event of an upward propulsion of the particles, one solution for trapping was the use an incident light coming from above (and not below as in the present work), as proposed in [16] for Silica-Gold Janus particles.

To sum up, our approach is applicable to a broad class of microswimmers, provided that both the laser parameters, and, to a lesser extent, the material properties of the particles are adequately selected to balance propulsive and optical gradient and scattering forces and trap the particle at the center of the beam. The effect of attractive gradient forces can be obtained by making sure that the swimmer is composed of a dielectric part with a (real) refractive index

higher than the medium (in our case water, see Supplementary Fig.5), and a beam size typically comparable or larger than the particle size. In our case, as mentioned in the main text, the trapping force on a single dielectric, TPM particle in the transverse plane typically compares to the propulsion force of the swimmer (approx 0.2 pN).

References

[1] T. Sugimoto, H. Itoh, T. Mochida, *Journal of Colloid and Interface Science* **205**, 42 (1998).

[2] M. Youssef, T. Hueckel, G.-R. Yi, S. Sacanna, *Nature Comm* **7**, 12216 (2016).

[3] A. Aubret, S. Ramananarivo, J. Palacci, *Current Opinion in Colloid & Interface Science* **30**, 81 (2017).

[4] C. van der Wel, *et al.*, *Langmuir : the ACS journal of surfaces and colloids* **33**, 8174 (2017).

[5] A. A. R. Neves, C. L. Cesar, *J. Opt. Soc. Am. B* **36**, 1525 (2019).

[6] S. Sukhov, A. Dogariu, *Reports on Progress in Physics* **80**, 112001 (2017).

[7] L. Rossi , *et al.*, *PNAS* **112**, 5286 (2015).

[8] J. R. Howse, *et al.*, *Phys. Rev. Lett.* **99**, 048102 (2007).

[9] I. Buttinoni, *et al.*, *Phys. Rev. Lett.* **110**, 238301 (2013).

[10] H.-R. Jiang, N. Yoshinaga, M. Sano, *Physical Review Letters* **105**, 268302 (2010).

[11] X. Peng, *et al.*, *Light: Science & Applications* **9**, 141 (2020).

[12] D. Palima, J. Gl?ckstad, *Laser & Photonics Reviews* **7**, 478 (2013).

[13] J. Liu, H.-L. Guo, Z.-Y. Li, *Nanoscale* **8**, 19894 (2016).

[14] J. L. Lawson, N. J. Jenness, R. L. Clark, *Optics express* **23**, 33956 (2015).

[15] O. Ilic, *et al.*, *Science Advances* **3** (2017).

[16] S. Nedev, *et al.*, *ACS Photonics* **2**, 491 (2015). PMID: 25950013.

[17] L. Lin, *et al.*, *Light: Science & Applications* **9**, 34 (2020).

[18] H. Moyses, J. Palacci, S. Sacanna, D. G. Grier, *Soft Matter* **12**, 6357 (2016).

[19] Aubret, A and Ramananarivo, S and Palacci, J, *Current Opinion in Colloid & Interface Science*, **30**, 81, (2017)

[20] Brown, A. and Poon, W. *Soft Matter* **10**, 4016 (2014).

[21] Palacci, J and Sacanna, S and Kim, S H and Yi, G R and Pine, D J and Chaikin, P M, *Philosophical Transactions of the Royal Society A: Mathematical, Physical and Engineering Sciences*, **372**, 20130372, (2014)

[22] Gomez-Solano, J. R. et al. *Scientific Reports* **7**, 14891 (2017).
