# Supplementary material for: Metamachines of pluripotent colloids
Source: Nat Commun. 2021 Nov 4;12:6398. doi: 10.1038/s41467-021-26699-6 (PMC8569212; doi:10.1038/s41467-021-26699-6)
Supplement: Supplementary file 2 — Description of Additional Supplementary Files. [file 41467_2021_26699_MOESM2_ESM.pdf]

Description of Additional Supplementary Files

File Name: Supplementary Movie 1
Description: **Re-programmable metamachines.** Self-propelling heterodimers are assembled into metamachines using patterns of optical traps. The traps uniquely serve to assemble the desired machine and are removed after initial templating, letting the machine to evolve autonomously. Spinning structures are first formed and merged to form a larger metamachine: a slender structure that autonomously rotates. The metamachine reconfigures following optical re-templating. Heterodimers in the metamachine reprogram autonomously and reorient, reversibly changing function. Turning off the blue light and removing the heterodimer activity, the system returns to equilibrium, the dynamics and interactions vanish. Heterodimers diffuse thermally and the metamachine breaks down. The successive templates are indicated in red at the bottom-right of the frames. The acceleration of the movie with respect to real time is indicated on the movie: sped-up 10 times for the formation of the substructures, and 2 times for the re-templating process.

File Name: Supplementary Movie 2
Description: **Activity-assisted optical trapping.** A static pattern of 31 optical traps forming the letters ”UCSD” is set on a field of view ≈ 104 µm2 . Active heterodimers self-propel with a persistent random walk leading to a diffusive behavior with effective diffusivity D ≈ 110 µm2 /s, 2 or 3 orders of magnitude above thermal diffusion. As they cross an optical trap, the heterodimers reorient, stand vertically, propelling against the substrate. The rapid diffusion of the self-propelled articles leads to rapid occupation (≈ 30 s) of the individual traps forming the letter ”UCSD”, avoiding the tedious pick-andplace required for optical trapping of microscopic objects. The movie is sped-up 2 times compared to real time.

File Name: Supplementary Movie 3
Description: **Chiral metamachines.** Centrosymmetric metamachines (assembly not shown but obtained using optical templates as shown previously) are merged to form a chiral metamachine. The chirality of the metamachine results from the conversion of particles lying flat on the edge into vertical particles as centrosymmetric metamachines merge. By controlling where the centrosymmetric metamachines meet and merge, we obtain two metamachines of opposite chirality and opposite rotation directions. Real-time movie.

File Name: Supplementary Movie 4
Description: **Translational metamachine.** Assembly of a translational metamachine by template and merge of centrosymmetric metamachines (assembly not shown but obtained using optical templates as shown previously) and heterodimers trapped individually. It highlights the role of the heterodimers in the concave region of the metamachine to introduce the forward/backward asymmetry and persistent self-propulsion of the machine. Real-time movie.

File Name: Supplementary Movie 5
Description: **Hybrid metamachines.** The design of metamachines is extended by inclusion of passive particles to the machine. Here, passive beads are trapped and surrounded by individual optical traps to form hybrid metamachines made of active heterodimers. They are further merged to form a large hybrid metamachine. Real time movie.
